# Considering Diastolic Dyssynchrony as a Predictor of Favorable Response in LV-Only Fusion Pacing Cardiac Resynchronization Therapy

**DOI:** 10.3390/diagnostics13061186

**Published:** 2023-03-21

**Authors:** Andra Gurgu, Constantin-Tudor Luca, Cristina Vacarescu, Lucian Petrescu, Emilia-Violeta Goanta, Mihai-Andrei Lazar, Diana-Aurora Arnăutu, Dragos Cozma

**Affiliations:** 1 Cardiology Department, “Victor Babes” University of Medicine and Pharmacy, 2 Eftimie Murgu Sq., 300041 Timisoara, Romania; 2 Research Center of the Institute of Cardiovascular Diseases Timisoara, 13A Gheorghe Adam Street, 300310 Timisoara, Romania; 3 Institute of Cardiovascular Diseases Timisoara, 13A Gheorghe Adam Street, 300310 Timisoara, Romania

**Keywords:** cardiac resynchronization therapy, LV-only fusion pacing, diastolic dyssynchrony, responders and super-responders

## Abstract

**Background:** CRT improves systolic and diastolic function, increasing cardiac output. Aim of the study: to assess the outcome of LV diastolic dyssynchrony in a population of fusion pacing CRT. **Methods:** Diastolic dyssynchrony was measured by offline speckle-tracking-derived TDI timing assessment of the simultaneity of E″ and A″ basal septal and lateral walls. New parameters introduced: E″ and, respectively, A″ time (E″T/A″T) as the time difference between E″ (respectively, A″) peak septal and lateral wall. Patients were divided into super-responders (SR), responders (R), and non-responders (NR). **Results:** Baseline characteristics: 62 pts (62 ± 11 y.o.) with idiopathic DCM, EF 27 ± 5.2%; 29% type III diastolic dysfunction (DD), 63% type II, 8% type I. Average follow-up 45 ± 19 months: LVEF 37 ± 7.9%, 34%SR, 61%R, 5%NR. The E″T decreased from 90 ± 20 ms to 25 ± 10 ms in SR with significant LV reverse remodeling (LV end-diastolic volume 193.7 ± 81 vs. 243.2 ± 82 mL at baseline, *p* < 0.0028) and lower LV filling pressures (E/E′ 13.2 ± 4.6 vs. 11.4 ± 4.5, *p* = 0.0295). DD profile improved in 65% of R with a reduction in E/E′ ratio (21 ± 9 vs. 14 ± 4 ms, *p* < 0.0001). Significant cut-off value calculated by ROC curve for LV diastolic dyssynchrony is E″T > 80 ms and A″T > 30 msec. **Conclusions:** The study identifies the cut-off values of diastolic dyssynchrony parameters as predictors of favorable outcomes in responders and super-responder patients with fusion CRT pacing. These findings may have important implications in patient selection and follow-up.

## 1. Introduction

Cardiac resynchronization therapy via biventricular (BiV) pacing is the standard of electrical care therapy that reduces ventricular dyssynchrony in advanced heart failure patients with complete left bundle branch block (LBBB) [1].

The presence of LV dyssynchrony contributes to aggravating LV hemodynamics, increases the risk of adverse cardiac events, and worsens the patient’s prognosis. In the case of BiV pacing, RV stimulation causes interventricular and intraventricular delays and therefore creates asynchrony [2]. The study of LV contraction in diastole could be an important tool for the assessment of the CRT response and the improvement of hemodynamics, which until now has not been intensively studied [3,4,5]. An elegant alternative to classical triple CRT that can maintain the RV activation synchronism, may be achieved using bicameral pacemakers with right atrium/left ventricule leads (RA/LV) in a carefully selected nonischemic CRT-P population with normal atrioventricular conduction [6,7]. However, less data is available concerning diastolic dyssynchrony and fusion pacing CRT. Most studies performed to identify CRT responders are focused on the analysis of parameters that reflect LV asynchrony during systole.

The aim of our study was to assess the outcome of LV diastolic asynchrony in a population of fusion pacing CRT without right ventricular (RV) lead. This article offers valuable data in a grey area of CRT and highlights the importance of LV dyssynchrony occurring in the diastolic phase.

## 2. Materials and Methods

### 2.1. Inclusion Criteria and Study Protocol

Prospective data were collected from a cohort of patients with idiopathic dilated cardiomyopathy and CRT-P indication. Briefly, inclusion criteria were heart failure New York Heart Association (NYHA) class III–IV despite optimal pharmacological treatment during the 3 months prior to CRT, QRS complex greater than 130 ms, typical LBBB pattern, preserved atrioventricular conduction. The exclusion criteria were represented by acute coronary syndrome or history of coronary artery disease, primary cardiomyopathy such as hypertrophic cardiomyopathy or stress cardiomyopathy (Takotsubo), secondary cardiomyopathies (e.g., sarcoidosis, amyloidosis, toxicity induced), permanent atrial fibrillation, severe comorbidities (e.g., renal, lung or liver failure, cerebral insufficiency, or terminal cancer) or noncardiac health conditions that limit physical activity (neuromuscular disorders, orthopedic conditions). Patients with CRT-D indication in primary or secondary prevention were also excluded.

Systematic 6-month full follow-ups (ECG, clinical, echocardiography), including exercise tests were recorded in all patients. Device and medication optimization were performed at each follow-up, when needed, in order to maximize CRT response [8,9].

### 2.2. Implantation Strategy

Permanent transvenous cardiac pacing was achieved using direct percutaneous subclavian vein puncture. The first pacing lead was positioned in the right atrium and the left ventricular lead was placed in different branches of the coronary sinus: anterolateral lateral, lateral, posterior, or posterolateral. In the case of atrioventricular block occurrence during implantation, a triple chamber CRT-P with right ventricular lead placement was available.

### 2.3. Transthoracic Echocardiography

Standard transthoracic echocardiography, including Doppler studies, was performed before and the day after device implant and at 6-month intervals using a Vivid E9 system (GE Health Medical, Milwaukee, WI, USA) and a 2.5 or 3.5 MHz phase array transducer. For each patient, ECG was simultaneously recorded. The following parameters were evaluated: left ventricular end-diastolic and end-systolic diameters (LVEDD, LVESD), left-ventricular end-diastolic and end-systolic volumes (LVEDV, LVESV), left-ventricular ejection fraction (LVEF) assessed by Simpson’s equation using the apical four-chamber view, the ratio of peak flow velocity in early diastole and peak flow velocity in late diastole during atrial contraction (E/A), deceleration time of E-wave (DTE), E/E’ ratio, LA diameter, area and volume (LAd, LAa, LAv), aortic root motion using M-mode. Myocardial PW Doppler velocity signals were reconstituted from TDI colour images, providing regional myocardial velocity curves.

**Figure 1:**
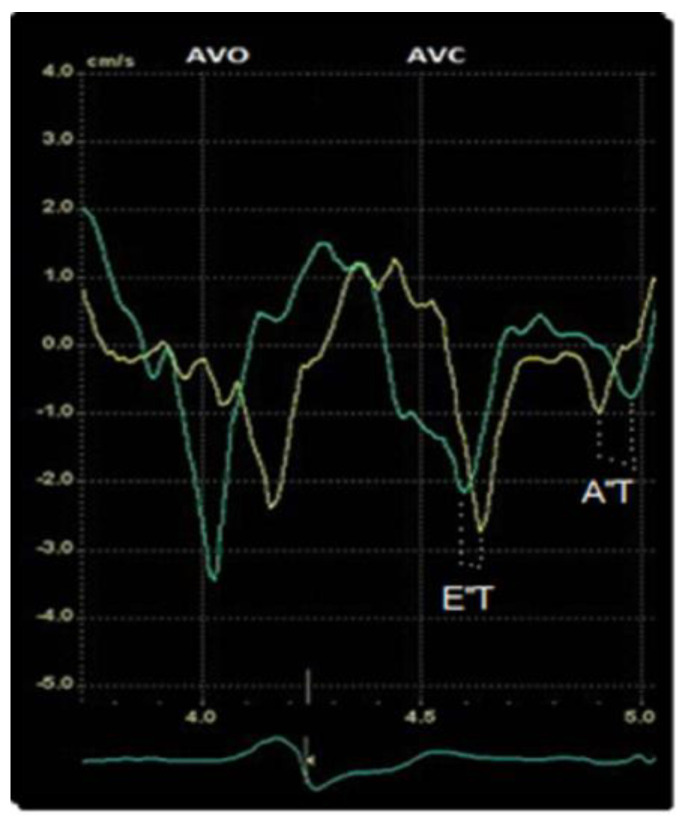
Offline speckle−tracking TDI timing assessment: the method used to measure E″T and A″T, green line = lateral velocity curve, yellow line = septal velocity curve.

Evaluation of diastolic dyssynchrony was performed in the diastolic phase by offline speckle-tracking-derived TDI timing assessment of the simultaneity of E″ and A″ basal septal and lateral wall in a 4-chamber view. In addition, we defined the following new parameters: E″ and A″ time (E″T/A″T) as the time difference between the septal and lateral wall E″ and A″ peaks (Figure 1). We measured these two new parameters outside ejective phase before and after resynchronization therapy. We performed a quantitative measurement in the time domain, which after CRT decreased, improving the diastolic filling time and therefore the mechanical efficiency and cardiac output.

### 2.4. CRT Response

Exercise tests, medication optimization, and device management were systematically performed to maintain constant fusion and improve CRT response. The following device check-up included 12-lead ECG pacing on/off, spontaneous AV interval, LV threshold, and response of pacemaker functioning during exercise. CRT assessment during ET analyzed maximum heart rate, 12-lead beat-to-beat ECG analysis of LV fusion pacing, occurrence of LV capture loss and improvement in exercise capacity. Based on the large clinical trials proving the clinical benefit of CRT, such as MIRACLE [10] or CARE-HF [11], we performed AV optimization for maximal diastolic filling time using PW Doppler echocardiography.

The CRT response was evaluated by means of 3 important criteria [12]: 1. clinical measures defined as improvement in NYHA functional class and quality of life, ET duration, and workload; 2. LV reverse remodeling (defined as >5% increase in LVEF, 15% decrease in LV end-systolic/diastolic volume and decreased mitral regurgitation degree); 3. outcomes measures defined as reductions in HF hospitalizations, morbidity, and all-cause mortality.

Three groups of patients were identified during the study: responders (R), super-responders (SR), and non-responders (NR). SR pts were defined as those with LVESV/LVEDV improvement ≥30% with an increase in diastolic filling time >50% and shorter E″T/A″T, while the responders were those with >5% increase in LVEF and 15% decrease in LVES/DV. Non-responder group was characterized by a reduction in LVESV/DV < 15% and larger E″T/A″T [8,12].

### 2.5. Statistical Analysis

Continuous data that approximate a normal distribution are presented as mean values ± standard deviation and as proportions for categorical variables. The T-test (or ANOVA if it was the case) was used for normally distributed continuous variables, and a chi-square test was used for categorical data. The optimal cut-off value of E″T and A″T was determined by receiver operating curve (ROC) analysis. Only variables with a *p*-value < 0.05 were considered to indicate statistical significance. All statistical analyses were performed with SPSS software version 23 and GraphPad software, version 9.5, Boston, MA, USA.

## 3. Results

The analytic group included 69 patients with idiopathic dilated cardiomyopathy implanted with a RA/LV DDD pacing system. During implantation, two patients developed acute AV block due to sheath manipulation, and they received a triple chamber CRT-P. Of all 69 initially assessed pts, 5 had poor echocardiographic images (bad alignment), and strain rate analysis was not possible with a final feasibility of 85%. The final true RA/LV CRT-P group consists of 62 patients.

The baseline demographic data are presented in Table 1. All devices were programed at a rest rate of 60 b/min and a maximum tracking rate (MTR) of 130 b/min. The atrioventricular interval, the key to success in obtaining and maintaining fusion stimulation, was programmed individually for each patient, based on surface electrocardiogram and using Doppler echocardiography to obtain maximal diastolic filling time. All patients had a QRS complex >130 msec, LBBB morphology with a mean QRS interval of 164 ± 18 ms and a QRS axis of −23 (±37) degrees. More than 75% of the patients had maximum pharmacological treatment at admission (maximum titration tolerated according to clinical and paraclinical variables). Taking into account that 35 patients (56%) were implanted before 2017, only a small number of patients received SGTL2 inhibitors and the fixed combination of sacubitril/valsartan at baseline (Table 1). During follow-up, SGTL2 inhibitors and sacubitril/valsartan were initiated in patients who persisted in the category of HfrEF or patients with associated type 2 diabetes mellitus and indication for SGLT2 inhibitors.

The average follow-up was 45 ± 19 months. Among the 62 patients, 29% had type III diastolic dysfunction (DD), 63% type II DD, and 8% type I DD.

After 6 months of RA/LV fusion CRT, among the 62 patients, there were 21 super-responders (34%), 38 responders (61%), and 3 non-responders (5%).The E″T decreased from 90 ± 20 ms to 25 ± 10 ms in SR with significant LV reverse remodeling (LV end-diastolic volume 193.7 ± 81 vs. 243.2 ± 82 mL at baseline, *p* < 0.0028) and lower LV filling pressures (E/E′ 13.2 ± 4.6 vs. 11.4 ± 4.5, *p* = 0.0295). A comparison between the baseline and the follow-up data is presented in Table 2.

The pulmonary artery systolic pressure (PASP) significantly improved in all patients. A reverse remodeling of the left atrium, correlated with its volume and surface, was noticed in both super-responders and responders. However, the responder patients had the maximum benefits in term of LA reverse remodeling. At baseline, severe mitral regurgitation (MR) was found in 12% of patients, 32% of patients had moderate MR, and 56% had mild MR. At the last follow-up, only the non-responders still had severe mitral regurgitation, and 20% of patients had moderate MR (8% SR and 12% R).

During follow-up, death occurred in 3 pts (2%) with type III DD, severe LA volume, and larger E″T/A″T (E″T > 85 msec A″T > 30 msec) due to refractory HF associated with pulmonary sepsis. At the end of the follow-up, NYHA class improvement was noted in 87% of patients: 89% in NYHA II (versus 10% at baseline) and 8% in NYHA III (versus 76% at baseline). Readmissions due to decompensated HF were noted in all NR patients (median readmission rate 3/year), while in R and SR groups only 6 patients (10%) needed hospitalization. The main causes for HF decompensation were diet and medication non-compliance, supraventricular tachyarrhythmias, and pulmonary infections.

**Figure 2:**
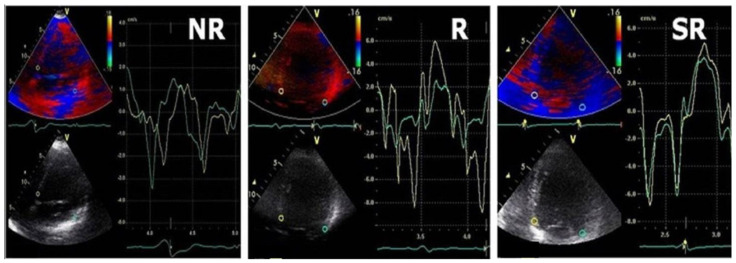
Typical TDI patterns in LV fusion pacing in SR/R/NR groups; green line = lateral velocity curve, yellow line = septal velocity curve.

**Figure 3:**
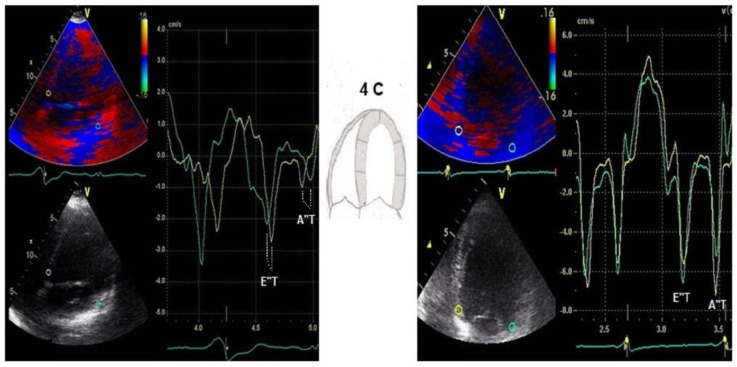
Example of a patient with baseline prolonged E″T and A″T and after 6 months of CRT; green line = lateral velocity curve, yellow line = septal velocity curve.

Among the baseline echocardiographic parameters in responders when compared with non-responders, DD profile improved in 65% of patients, reaching a significant statistical importance in E/E′ ratio both in super-responders and in responders. Both E″T and A″T were significantly decreased in responders when compared with baseline (76 ± 13 vs. 51 ± 11 and 26 ± 8 vs. 7 ± 5), and the non-responders group was associated with larger baseline E″T and A″T and no statistically significant difference was noted in these patients after CRT. (Figure 2 and Figure 3).

### Prediction of Response

**Figure 4:**
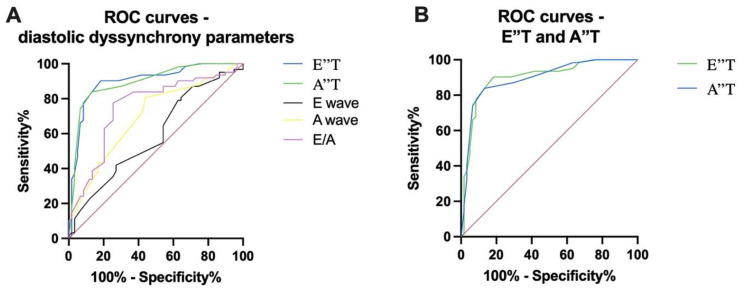
(**A**) ROC (receiver operating characteristic) curves analyzing the most important parameters of diastolic asynchrony: E wave, A wave, E/T ratio, E″T, and A″T. (**B**). ROC curves for E″T and A″T parameters.

We assessed the most important parameters of diastolic asynchrony that predict the response to CRT using ROC (receiver operating characteristic) analysis. Thus, for E wave an area under the ROC curve (AUC) of 0.5890 (95% confidence interval 0.4874 to 0.6905, *p* = 0.0914) was recorded; for A wave, an AUC of 0.6968 (95% confidence interval 0.6033 to 0.7904, *p* = 0.0002) was recorded; and for the E/A ratio, an AUC of 0.7523 (95% confidence interval 0.6628 to 0.8418, *p* < 0.0001) was recorded. Compared to these parameters, a value of E″T > 80 ms (AUC = 0.8989, 95% confidence interval 0.8401 to 0.9577, *p* < 0.0001) and a value of a A″T > 30 ms (AUC = 0.8938, 95% confidence interval 0.8351 to 0.9526, *p* < 0.0001) were shown to be stronger predictors for the response to CRT. These comparisons are highlighted in Figure 4.

## 4. Discussion

Most studies performed to identify CRT responders are focused on the analysis of parameters that reflect LV asynchrony during systole. In our study we identified the cut-off values of newly defined diastolic dyssynchrony as a predictor of favorable outcomes. Furthermore, we highlight the importance of LV dyssynchrony occurring in the diastolic phase.

Cardiac mechanical efficiency requires synchronous wall motion in the same phase of the cardiac cycle, and the longer the period of asynchronous contraction and relaxation, the greater the net mechanical impairment [13]. Measurement of regional myocardial electro-mechanical events with two-dimensional strain imaging by speckle tracking and TDI-derived strain imaging facilitates identification of mechanical, and they proved to be useful tools both for the evaluation of CRT response and for the selection of patients who might benefit from cardiac resynchronization therapy [14,15,16,17,18,19].

Several TDI parameters were proposed, generally using the difference in time to the peak of systolic sustained velocity. Although this method could provide several details on diastolic ventricular function, the studied parameters were focused exclusively on the dyssynchrony in the ejection phase [20,21,22,23]. Soogard et al. performed a pioneering investigation using tissue tracking techniques and demonstrated that the number of segments displaying longitudinal contraction during diastole is directly proportional to the severity of dyssynchrony [3]. However, this method could not evaluate the degree of temporal delay. Otherwise, Porciani et al., using strain imaging, measured the total amount of time spent by the 12 segments contracting after aortic valve closure, reflecting the importance of LV dyssynchrony occurring in the diastolic phase [5].

In our study, we highlight the importance of evaluating diastolic asynchronism. We defined two new parameters (E″T and A″T) as the time difference between the septal and lateral wall E″ and A″ peaks, respectively. We measured these parameters before and after resynchronization therapy. In the majority of SR and R patients we observed a substantial reduction of E″T and A″T after implantation, which indicates that a quantitative measurement in the time domain could predict LV functional recovery. Therefore, they are indirect markers of systolic asynchrony.

As is well known, poor reprogramming of AV delays contributes to the reduction of the effectiveness of CRT [24,25,26]. The key target of programming has been to deliver 100% of ventricular capture in order to achieve the maximal outcomes [7,27,28].

To maintain constant fusion in our population, we have performed AV optimization using PW Doppler echocardiography with assessment of the mitral inflow pattern, drug optimization, and CRT reprogramming during follow-ups with exercise tests. Hereby, we noticed that a prolonged diastolic filling time was associated with better diastolic synchronization and therefore a reduction in E″T and A″T. In addition, it must be emphasized that in our study we have provided exclusive fusion pacing CRT using RA/LV dual chamber devices, without the potential of additional desynchronization through right ventricular pacing. Our analysis was limited by collecting data from only one image plane, with the apical view providing in all our patients reliable information on radial and longitudinal deformation in two opposite walls. Although we included in the study only patients with non-ischemic cardiomyopathy, typical LBBB, and QRS > 130 ms (well-known predictors of favorable CRT response), the patients are different in terms of comorbidities and HF treatment, and this reflects a real-life population of patients.

The low number of non-responders is a significant limitation of the study that can reduce the impact of the statistical test. However, the small number of non-responders is due to a highly selective population included in the study and a close and tailored follow-up for each patient in order to maximize the CRT response. To the best of our knowledge, there is no validated data regarding this subject, and we hope that the new introduced parameters will be analyzed in larger, randomized trials with higher statistical power.

Demonstrated in several studies, approximately 30% of patients will be CRT non-responders [29,30]. Among the main causes identified are lower LVEF, longer HF history, higher pulmonary artery pressure, suboptimal AV timing, and lower BIV percentage. Our data showed that NR pts were associated with type III DD, elevated E/E’, severe LA volume and prolonged E″ and A″ times, which remained before and after CRT therapy. Furthermore, a post-systolic shortening, considered by some authors to be an expression of myocardial asynchrony [5,31,32,33], was noticed in the group of NR patients. An asynchronous contraction after LV systolic ejection can occur in different conditions, such as LV overload [31]. This allows us to conclude that elevated filling pressures are directly proportional to E″T/A″T, parameters that predict LV reverse remodeling.

Although the end point of the study was not a clinical one in terms of mortality or HF hospitalizations, we succeeded in demonstrating that E″T and A″T have powerful implications for predicting CRT response.

## 5. Conclusions

In conclusion, this study identifies the cut-off values of new defined diastolic dyssynchrony parameters as predictors of favorable outcomes in responders and super-responder patients with fusion CRT pacing. These findings may have important implications in patient selection and follow-up. Larger randomized trials are needed to define the role of diastolic asynchronism in cardiac resynchronization therapy.

## Figures and Tables

**Table 1:** Baseline demographic data.

		All Patients (*n* = 62)	SR (*n* = 21)	R(*n* = 38)	NR (*n* = 3)
Age, years (mean ± SD)		62 ± 11	61 ± 9	69 ± 10	73 ± 11
Male, *n* (%)		38 (61%)	11 (52%)	25 (65%)	2 (66%)
NYHA functional class, *n* (%)	II	6 (10%)	2 (10%)	4 (11%)	0
	III	47 (76%)	18 (86%)	27(71%)	2 (67%)
	IV	9 (1%)	1 (5%)	7 (18%)	1 (33%)
NT-proBNP pg/mL, *n* (%)	400–1000 pg/mL	11 (17%)	3 (14%)	8 (21%)	0
	1000–2000 pg/mL	36 (58%)	15 (71%)	20 (52%)	1 (33%)
	>2000 pg/mL	16 (26%)	4 (19%)	10 (26%)	2 (66%)
Hypertension, *n* (%)		35 (56%)	11 (52%)	21(55%)	3 (100%) *
Diabetes mellitus, *n* (%)		25 (40%)	9 (43%)	14 (37%)	2 (67%)
Dyslipidemia, *n* (%)		48 (77%)	19 (90%)	26 (68%)	3 (100%) *
Obesity, *n* (%)		26(42%)	10 (47%)	14 (36%)	2 (67%)
Chronic kidney disease, *n* (%) **		25 (40%)	5 (23%)	17 (44%)	3 (100%) *
Medication	Beta blockers, *n* (%)	46 (74%)	18 (85%)	26 (68%)	2 (67%)
	Ivabradine, *n* (%)	26 (42%)	9 (43%)	15 (39%)	2 (67%)
	ACEI/ARB, *n* (%)	54 (87%)	19 (90%)	33 (86%)	2 (67%)
	Diuretics, *n* (%)	56 (90%)	18 (85%)	35 (92%)	3 (100%)
	Mineralocorticoid receptor blockers, *n* (%)	47 (76 %)	14 (66%)	31 (81%)	2 (67%)
	Sacubitril/valsartan, *n* (%)	8 (12%)	2 (9%)	5 (13%)	1 (33%)

* *p* value < 0.05. ACEI = angiotensin-converting-enzyme inhibitors, ARB = angiotensin receptor blockers, *n* = number, SD = standard deviation. ** Chronic kidney disease is defined as a reduction in creatinine clearance <90 mL/min. None of the patients in our cohort had creatinine clearance <30 mL/min.

**Table 2:** Conventional and tissue Doppler echocardiographic parameters of super-responders, responders, and non-responders at baseline and at 6-month follow-up.

	Super-Responders		*p*	Responders (*n* = 38)		*p*	Non-Responders		*p* Value
	(*n* = 21)						(*n* = 3)		
	Baseline	FU		Baseline	FU		Baseline	FU	
**EF (%)**	31 ± 4	45 ± 4	<0.0001	26 ± 5	34 ± 6	<0.0001	24 ± 4	26 ± 5	0.61
**LV EDV (mL)**	243 ± 82	193 ± 81	0.0028	272 ± 72	217 ± 64	0.0007	308 ± 105	278 ± 93	0.72
**LV ESV (mL)**	126 ± 44	75 ± 21	<0.0001	206 ± 62	148 ± 51	<0.0001	224 ± 73	174 ± 29	0.33
**LA Volume (mL)**	92 ± 30	89 ± 30	0.74	116 ± 40	101 ± 33	0.07	148 ± 33	141 ± 0.5	0.73
**LA Surface (cm^2^)**	24 ± 4	22 ± 5	0.16	30 ± 7	27 ± 7	0.06	32 ± 2	33 ± 3	0.70
**PASP (mmHg)**	37 ± 12	31 ±8	0.06	42 ± 13	34 ± 10	0.0036	58 ± 6	38 ± 6	0.0151
**E/E′**	13 ± 4	11 ± 4	0.0295	21 ± 9	14 ± 4	<0.0001	29 ± 4	23 ± 7	0.26
**E/A**	2 ± 0	1 ± 0	−	1.4 ± 5	0.8 ± 3.9	0.44	2 ± 0	2 ± 0	−
**E′ Time (ms)**	90 ± 20	25 ± 10	<0.0001	76 ± 13	51 ± 11	<0.0001	118 ± 10	102 ± 14	0.18
**A′ Time (ms)**	16 ± 7	8 ± 5	=0.0001	26 ± 8	17 ± 5	<0.0001	67 ± 9	57 ± 5	0.16

All data is presented as mean ± standard deviation. EF = ejection fraction, FU = follow-up, LV EDV = left-ventricle end-diastolic volume, LV ESV = left-ventricle end-systolic volume, LA = left atrium, PSAP = pulmonary artery systolic pressure.

## Data Availability

The data presented in this study are available on request from the corresponding author. The data are not publicly available due to ethics and data security reasons.
